# Increasing Impact Strength of a Short Glass Fiber Compression Molded BMC by Shortening Fibers without Change in Equipment

**DOI:** 10.3390/ma15031145

**Published:** 2022-02-01

**Authors:** Michael C. Faudree, Yoshitake Nishi, Michelle Salvia

**Affiliations:** 1 Faculty of Liberal Arts and Science, Tokyo City University, Yokohama-shi 224-8551, Japan; 2 Graduate School of Engineering, Tokai University, Hiratsuka-shi 259-1292, Japan; west@tsc.u-tokai.ac.jp; 3 Kanagawa Institute of Industrial Science and Technology (KISTEC), Ebina-shi 243-0435, Japan; 4 Ecole Centrale de Lyon, Ecully, CEDEX, 69134 Lyon, France; michelle.salvia@ec-lyon.fr

**Keywords:** bulk molding compound, polyester, glass fibers, Charpy impact, solidification texture angle

## Abstract

Bird strike, volcanic rock, hailstones, micrometeoroids, or space debris can cause damage to aircraft and space vehicles, therefore their composite materials must have high impact resistance to maximize safety. In a 55% wt. CaCO_3_ compression molded short glass fiber polyester GFRP-BMC (bulk molded compound), shortening the nominal 6.4 mm fiber length formulation, by 30 min extended mixing, to 0.44 mm was found to increase Charpy impact values, *a*_uc_, without a change in the compression molding equipment. Specimens were cut from square panels in a spiral configuration in conformity with ASTM D 6110-02 for orthotropic panels, the flow direction approximately radially outward from the charge. At a median-fracture probability of *P*_f_ = 0.50, extended mixing improved *a*_uc_ by 29%, from 7.43 to 9.59 kJm^−2^, and for each solidification texture angle, namely, 0 to 90 (random), 71, 45 and 18 deg, the *a*_uc_ increased by 25% (6.26 to 7.86 kJm^−2^), 18% (9.36 to 11.07 kJm^−2^), 35% (7.68 to 10.37 kJm^−2^), and 20% (6.96 to 8.36 kJm^−2^), respectively. This strengthening can be explained by an increased number of thermal compressive stress sites between the glass fiber and matrix due to a difference in the coefficient of thermal expansion (CTE) during cool-down, and shrinkage, with an increased number of spaces between fibers, |*S*_f_| from 217 to approximately 2950 per mm^3^, enhancing impact energy.

## 1. Introduction

Bulk molded compounds (BMCs) are utilized for non-heavy load bearing parts, having advantages over metals in being lightweight, lowering fuel consumption for aircraft and other vehicles to reduce CO_2_ emissions, and are also corrosion resistant. BMCs typically contain ~5 to 30 wt.% fiber and are highly filled [1,2,3,4,5,6,7,8,9,10]. Glass fiber reinforced polymer GFRP-BMCs contain ~5 to 30 mass% chopped fiber about 3 to 13 mm in length [1,3,7], and are highly filled with CaCO_3_ powder ranging from ~35 to over 50 mass% [7,8], while other fillers can include TiO_2_, Al_2_O_3_, SiC, Mg(OH)_2_, ZnO, [9] fumed silica [10], fly ash [5], or waste thermosetting BMC [2], to name a few [1,2,3,4,5,6,7,8,9,10].

Previously, unexpected findings for FRP composites, namely that of polyester with styrene-butadiene copolymer reinforced with glass fibers and CaCO_3_ filler, were reported where the tensile modulus could be increased by 5 to 25% [8] along with fracture strength, and its strain increased ~60 and ~40% [7], by decreasing mean fiber length from the commercial 6.4 mm (1/4 in) to 0.44 mm via 30 min of extended mixing of paste prior to injection molding, without a change in the injection molding equipment. This was a new finding not observed prior, because most of the literature concerns 2-phase fiber and polymer composites whose mechanical properties, such as impact strength, tensile stress and strain, increase with increasing fiber length [11,12,13,14,15,16,17], and longer fibers are reported as desired [18]. However, the GFRP-BMC is a 3-phase filler, fiber and polymer system in which the CaCO_3_ filler particles appear to play a role in strengthening the composite when shortening the fiber length below that of commercial length [7,8]. Therefore, this study presents new experimental results that impact strength and can be increased by a 30 min extended mixing in the GFRP-BMC.

The strengthening method is congruent to earlier studies [7,8] of 30 min extended mixture of commercial 6.4 mm fiber length GFRP-BMC paste to shorten glass fibers to 0.44 mm, prior to injection molding [7]. Nominal fiber length of 6.4 mm was chosen since it was commercially available, whereas the 0.44 mm length was produced by 30 min of mixing [2]. The 0.44 mm fiber length was determined by measuring several hundred fibers of polished masticated samples by SEM, showing a mean fiber length of 0.44 mm (standard deviation = ± 0.203 mm) [1,7]. Generally, two standard deviations equal about 95% of the population (0.04 mm < *l*_fiber_ < 0.85 mm), representing a wide distribution from the extended mixing, yet significantly less than 6.4 mm [1,7], and below the reported critical fiber length for GFRPs of ~1.0 mm (0.56–0.59 mm for nylon GFRP; 1.4 mm for polypropylene (PP) GFRP; and 0.68 to 0.84 mm for polybutylene terephthalate (PBI) GFRP) [14]; however these critical lengths are for 2-phase systems [14,15] and would depend on the strength of the coupling. Nevertheless, the glass fibers have a strong coupling agent to adhere to the polymer component in the GFRP-BMC. Therefore, mean fiber lengths of the 6.4 and 0.44 mm data sets are considered to have enough variance for the results to be reliable [7,8]. With a 30 min extended mix, it is assumed, in the compression molded samples, that fiber length is reduced to approximately 0.44 mm. The CaCO_3_ filler (<1 to ~7 μm) is reported to be unaffected by 30 min extended mixing [2].

**Figure 1:**
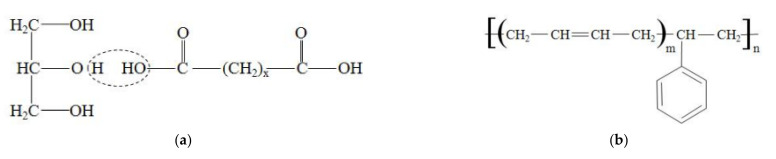
Rational formulae of (**a**) thermoset polyester, and (**b**) styrene butadiene.

The polymers used in the formulation are thermoset polyester, with styrene-butadiene, as illustrated in Figure 1.

To provide background, fiber length studies on polymer composites appear to be predominantly limited to 2-phase fiber and polymer systems, where mechanical properties are reduced with shortened fibers [11,12,13,14,15,16,17,19,20]. In a study of polypropylene GFRP at a fiber weight percent between 3 and 60%, varying fiber length from 0.1 to 50 mm showed that stiffness was reduced at fiber lengths under 0.5 mm, and was nearly unaffected above 0.5 mm [11]. High weight percentages above 40% were reported to cause fiber packing problems, with an increase in void formation reducing the modulus [11]. It was also reported for polypropylene GFRP, that impact properties were raised as fiber length was increased to 6.4 mm, while a strain energy model predicted 8 mm as the optimal fiber length [13]. For short carbon fiber polypropylene CFRP, Izod impact, Rockwell hardness, tensile strength and modulus, and flexural strength and modulus values were lowered as fiber lengths were shortened: 10 → 5 → 2 → 1 → 0.5 mm [17]. Similarly, Capela et al. found in compression molded CFRP with Biresin^®^CR120 resin, for higher *V*_f_ of 60 wt.% carbon fibers, the optimum fiber length for tensile properties was 4 mm. Stiffness and tensile strength increased by increasing fiber length from 2 mm to 4 mm, but then decreased for 6.4 mm fibers. The decrease was attributed to poor fiber dispersion, and disorder within the matrix with the longer fibers [19].

In addition, a trend of decreasing mechanical properties with shorter fiber length has been found for green composites (2-phase). In hemp fiber reinforced thermoplastic polyurethane, increasing the fiber length from 6 to an optimum of 15 mm increased tensile strength from 16 to 27 MPa, while a further increase to 40 mm resulted in little change [20].

Because assessing the effect of fiber length can be difficult, many studies on 2-phase GFRP systems have utilized numerical modelling to characterize the increase in mechanical properties as fibers are lengthened, predicting that shortening fibers below the critical length of ~1.0 mm greatly reduces mechanical properties [14,15].

Conversely, we demonstrate a new result not previously reported in the literature, that for the highly-filled 3-phase GFRP-BMC, impact values can be increased by shortening fibers below 1.0 mm. The strengthening mechanism is similar to ceramics, cemented carbide composites, and metal matrix composites (MMC), in that strength is increased by decreasing the particle size [21,22,23]. For instance, in WC-Ni cemented carbide composites, strength was increased for 0.5 μm particles above the coarser 1.7 μm particles [21]. In Al_2_O_3_ dispersed ceramics, the smaller 16 μm diameter particle size raised fracture stress 30% above 41 μm diameter particle size [22]. The enhancements were attributed to the difference in the proliferation of residual stress sites by CTE (coefficient of thermal expansion) between particles and matrix, as particle size was reduced. Reported SEM observations for injection molded polyester GFRP-BMC showed CaCO_3_ filler particles to be <1 to 7 μm [8], about the same size as reported for ceramics, namely, from <1 μm to several microns [21,22,23].

As for the effect of percent filler on BMC, few studies were found. One study was performed with polymer mixed with a filler of waste thermosetting BMC (filler, glass fiber and polymer) crushed into a powder, where filler amounts of 0 → 30 → 40 → 50 → 60 wt.% resulted in fairly low impact values of 4.91 → 1.76 → 1.69 → 1.73 → and 1.93 kJm^−2^, respectively [2]. Shore D hardness was virtually unchanged at ~66 ^o^Sh D [2]. Loss of properties were attributed to poor adhesion between filler and matrix. Since our study focuses on fiber length, the effect of percent filler will not be covered here.

For 3-phase GFRP-BMCs, decreasing mechanical properties with decreasing fiber length has been reported [3]. In a highly filled 50 wt.% CaCO_3_ GFRP-BMC with ~30 wt.% polymers and 16.7 wt.% glass fibers, bending strength and modulus were reported to decrease with glass fiber length. For 12.7 → 6.4 → 3.2 mm (1/2 → 1/4 → 1/8 in) fibers, bending strength decreased: 115 → 81 → 53 MPa, along with bending moduli: 13.4 → 12.3 → 11.5 GPa [3]. This indicates that choice of binder and other additives are important for good adhesion of matrix with filler and fibers in GFRP-BMCs.

In our study, the CTE of cured polyester resin matrix (55 to 100 × 10^−6^/K) [24] is approximately ten times higher than E-glass fibers (5.4 × 10^−6^/K) [25], hence, when the panels are cooled, the matrix will volumetrically contract onto the fibers more than the fibers contract themselves, creating compressive residual stresses onto the fibers. It follows that shorter fiber length creates a higher number of spaces between fibers (*S*_f_,) [8] allowing increased sites for action of CTE difference to collectively stiffen the composite, thereby raising impact strength.

In fact, tensile tests of GFRP-BMC showed that shortening glass fibers: 6.4 → 3.2 → 0.44 mm increased stiffness in the form of initial tensile modulus (d*σ*/d*ε*)_o_ (strain, *ε* = 0 to 0.05%) with decreasing fiber length of 6.19 → 7.18 → 7.86 GPa [8]. Maximum moduli (d*σ*/d*ε*)_max_ between the zero point and Δ*ε* = 0.4% were also increased: 7.50 → 8.86 → 9.54 GPa for the 6.4 → 3.2 → and 0.44 mm samples, respectively. In sum, shorter 0.44 mm samples exhibited a 27% and 40% increase in (d*σ*/d*ε*)_o_ and (d*σ*/d*ε*)_max_, respectively, over those of commercial 6.4 mm [8]. An increase in modulus with lowering fiber length was attributed to increasing spaces between fibers acting with a CTE difference as mentioned above [8]. Interestingly, the 0.44 mm samples appeared to show evidence of slight strain hardening during early tensile deformation. Although an earlier study of the data showed little change in modulus with decreasing fiber length [7], when analyzed in more detail at strain increments Δ*ε* = 0.00734%, the increase was found [8].

As for higher deformations with increasing damage, tensile stress–strain curves of the GFRP-BMC exhibited ~60 and −40% increase in tensile fracture stress and strain, respectively, by shortening fibers from a commercial length of 6.4 mm, to 0.44 mm [7]. Acoustic emission (AE) detected three times the number of cracks, while scanning electron microscopy (SEM) showed increased fiber debonding at fiber ends and along fiber lengths in the 0.44 mm samples compared with the 6.4 mm sample. Increases in tensile properties were attributed to strain fields from the fiber debonding proliferating expansion sites, which have been found to halt cracks before their critical length is reached [7]. However, damage from impact occurs much faster than tensile tests, therefore, it would seem the cracking dynamics of sudden impact would differ from that of tensile. It follows that the stress–strain curves showed a higher modulus at all strains throughout tensile deformation up to fracture [7], therefore, an increase in impact resistance seems mostly due to increased stiffness, and less to expansion around fibers caused by debonding. Moreover, since the impact tip hitting the specimen is a straight line across the specimen thickness, a higher number of fibers would be directly impacted in the higher-dispersed 0.44 mm samples, in which hardness is more evenly distributed. Therefore, the main mechanism of increasing impact resistance in the GFRP-BMC by 30 min of extended mixing appears be from an increase in stiffness throughout the impact process.

Unlike 2-phase fiber and polymer systems, within the matrix itself, the third phase of CaCO_3_ particles evidently assists in strengthening [8]. Particles well-dispersed and at short distances from each other can be advantageous in maximizing residual thermal stresses [26] leading to the design of stronger BMC composites. Within spaces between fibers, *S*_f_, the filler and polymer sub-system undergoes compressive stresses as a whole, the resin shrinking around CaCO_3_ filler and fibers, with higher and more dispersed force than 2-phase fiber and polymer systems, increasing—not decreasing—mechanical properties as fiber length is decreased.

To the knowledge of the authors, increasing the impact strength of GFRP-BMC by shortening fibers has not yet been reported. Therefore, the goal is to demonstrate that Charpy impact values can be increased in compression-molded short-fiber GFRP-BMC panels containing a high percentage (55 mass%) of CaCO_3_ filler, by shortening fibers from a commercial length of 6.4 mm to 0.44 mm, by 30 min extended mixing prior to molding, without changing the compression molding equipment.

## 2. Materials and Methods

### 2.1. Preparation of GFRP

The components, molding parameters, and fiber lengths of the GFRP-BMC compression molded panels [27,28,29] are summarized in Table 1, Table 2 and Table 3.

Panels were provided by Premix, Inc., (now Citadel) of North Kingsville, Ohio, with nominal 6 mm to 6.4 mm chopped glass fibers (exact nominal length between 6 and 6.4 mm proprietary), reported here as U.S. 1/8 in (6.4 mm) [7,27]. As mentioned above, the mean fiber lengths of the 6.4 and 0.44 mm data sets are considered to have enough variance for results to be reliable [7,8]. Optical microscope Nikon Eclipse ME600 was used to examine the polished GFRP-BMC samples.

**Figure 2:**
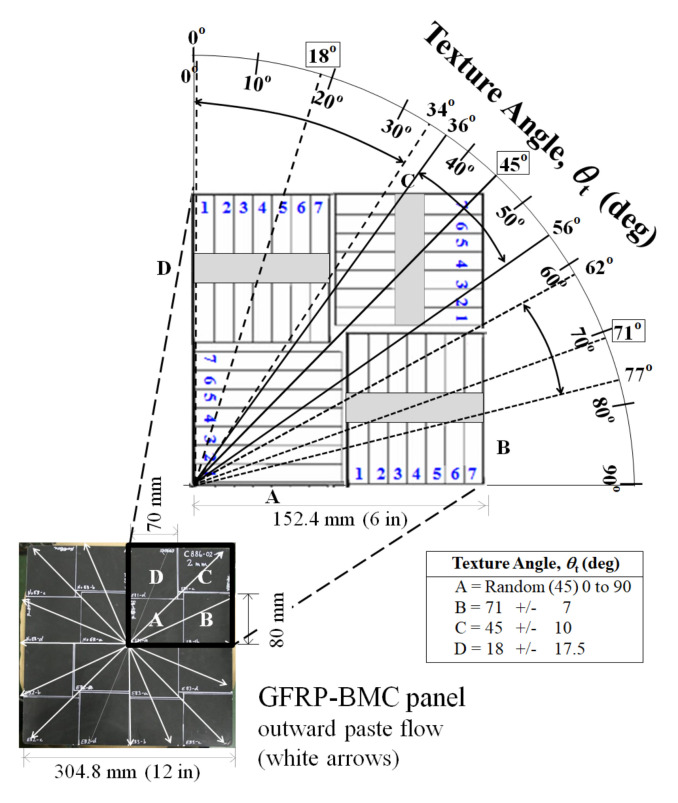
GFRP-BMC panel and specimen cutting configuration according to ASTM D 6110-02, taken from Faudree et al. (2018) [28].

Figure 2 shows Charpy impact samples with dimensions 80 × 10 × 2 mm cut in a spiral formation according to ASTM D 6110-02 (2002) [29], since mold flow in the compression molded GFRP-BMC panels is anisotropic. This resulted in four 7-sample sub-quadrants designated “A, B, C, or D” according to solidification texture angle with respect to the long 80 mm direction, *θ*_t_ (deg), of: random (45) 0 to 90; (71 ± 7); (45 ± 10); and (18 ± ~17.5) [27,28].

Table 4 shows 56 tested samples of GFRP-BMC for each of the two fiber length sample data sets of 6.4 and 0.44mm, divided into 14 samples within each section A, B, C, and D, respectively. Figure 2 shows the location of the sample in the panel, according to the sample number, which is always counted from center 1 to 7. These were designated as “A1, A2, A3; B1, B2, B3, etc.”. Since 2 sub-quadrants of 7 samples each were tested, to distinguish the samples, the second quadrant was designated as “A1′, A2′, A3′; B1′, B2′, B3′, etc.”, therefore, the same location according to the solidification flow angle can be compared.

### 2.2. Charpy Impact Tests

**Figure 3:**
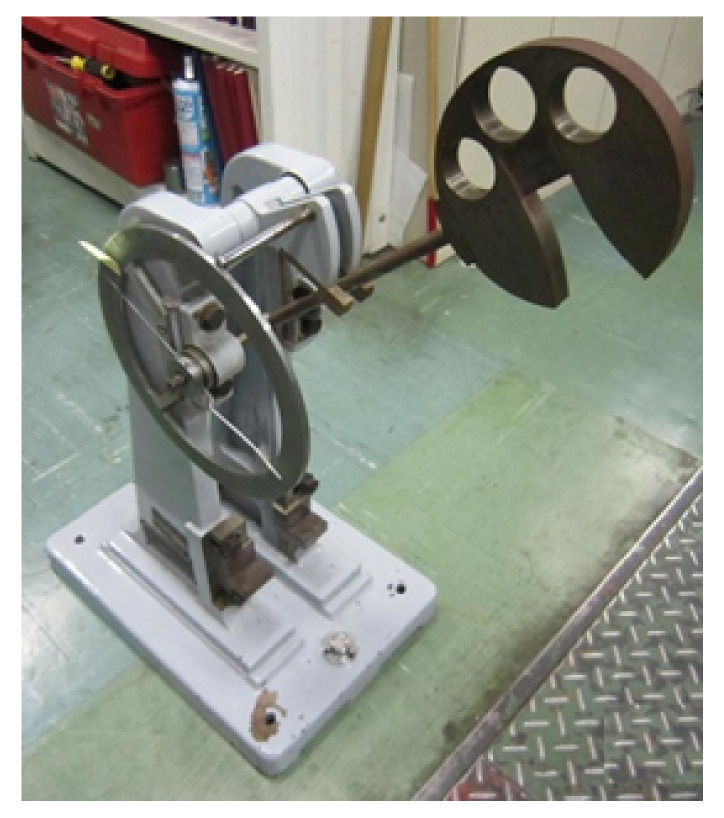
Charpy impact tester.

Charpy impact tests were conducted to evaluate fracture toughness, *a*_uc_ (kJm^−2^), of the GFRP-BMC samples. The Charpy impact test is a quick and easy method often employed to evaluate the safety of materials for quality control (QC). Figure 3 shows the apparatus used (Shimadzu Corp. No. 51735) conforming to JIS K 7077-1991 testing standard, which operated by a drop weight pendulum [27,28,29,30,31,32,33]. A diamond cutter (MC-201, MARUTO) was used to cut unnotched specimens to size according to JIS K 7077-1991 [30].

Impact fracture energy, E (kJ) is calculated in Equation (1):

*E* = *WR*[(cos *β* − cos *α*) − (cos *α*’ − cos *α*)(*α* + *β*)/(*α* − *α*’)] (1)

where *W* = hammer mass (0.86 kg), *R* = distance from impact point of specimen to rolling center (0.21 m), *β* = finish angle after impact (radians), *α* = start angle (2.3 radians) and *α*’ = average angle of 3 blank tests for calibration [30,31]. The *a*_uc_ (kJ/m^2^) is calculated by [30,31]:

*a*_uc_ = *E*/(*bt*) (2)

where *b* = sample width (~10 mm) and *t* = thickness (~2 mm). When the specimen was placed in the holder, there was a gap distance of 40 mm. The fracture probability, *P*_f_, is expressed using the median rank method [33]:

*P*_f_ = (*I* − 0.3)/(*N*_s_ + 0.4) (3)

where *N*_s_ is the total number of samples (*N*_s_ = 56 for each data set; or 14 for each sub-quadrant, A, B, C, D) and *I* is the ascending strength order of each sample, respectively.

## 3. Results and Discussion

### 3.1. Effect of Shortening Fibers on Impact Strength of Panel at All Texture Angles, θ_t_ Cumulative, and Sub-Quadrants

Table 5 shows the results for all texture angles, *θ*_t_ cumulative. Shortening fibers from the commercial length of 6.4 mm to 0.44 mm raised the average *a*_uc_ by 26% from 7.63 to 9.62 kJm^−2^ in the compression molded GFRP-BMC panels (standard deviation in brackets). Moreover, the *a*_uc_ was increased by 27.6, 19.1, 29.9 and 29.5% for each sub-quadrant A, B, C, D, respectively, demonstrating that *a*_uc_ can be raised regardless of the texture angles examined in the panel.

**Figure 4:**
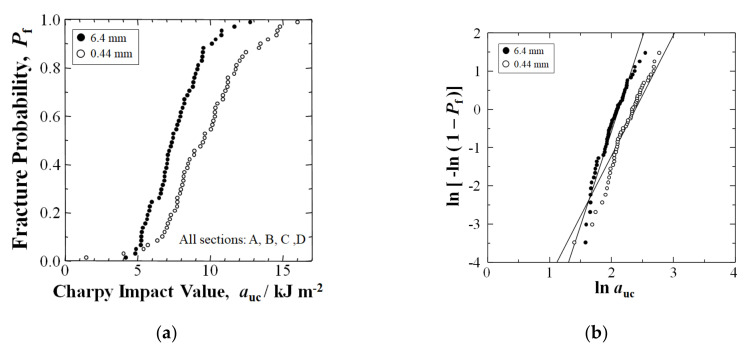
(**a**) Relationship between Charpy impact value, auc, and fracture probability, Pf, for nominal 6.4 mm and extended mixing 0.44 mm samples in a GFRP-BMC compression-molded panel, with the cumulative of Sections A, B, C and D equaling 56 samples each of the 6.4 and 0.44 mm fiber length sample data sets, respectively; (**b**) Weibull 2-parameter plots of data in (**a**).

Figure 4a shows the *P*_f_ vs. *a*_uc_ plot for all solidification texture angles, *θ*_t_, of all individual samples of Sections A, B, C, and D, cumulatively in the two 56-specimen data sets, namely, the 6.4 mm and the shortened fiber 0.44 mm samples, respectively. At median-fracture probability, *P*_f_ = 0.50, therefore, shortening fibers by 30 min extended mixing improved the Charpy impact value *a*_uc_ by 29%, from 7.43 to 9.59 kJm^−2^. Moreover, at high *P*_f_ = 0.88, *a*_uc_ improved by 40%, from 9.57 to 13.38 kJm^−2^. The *a*_uc_ was improved at all *P*_f_ above 0.03.

### 3.2. Weibull Analysis: All Texture Angles, θ_t_ Cumulative

Weibull analysis is a standard method widely utilized to compare many structural materials [34,35,36]. The 2-dimensional Weibull coefficient (*n*) is calculated from the experimental Charpy impact values (*a*_uc_) and fracture probability (*P*_f_), where (*a*_uc_/*a*_o_) is the rupture risk [34,35,36]:

*P*_f_ = 1 − exp[−(*a*_uc_/*a*_o_)*^n^*] (4)

The linear form is [34,35,36]:

ln(−ln(1 − *P*_f_)) = *n*[ln *a*_uc_ − ln *a*_o_] (5)

Figure 4b shows the resulting Weibull plots for the 6.4 mm and 0.44 mm data sets, respectively, where the *n* values are slope lines. In the 0.44 mm data set, although *n* reduced from 4.92 to 3.15, the impact values increased by 29% at a median–*P*_f_ of 0.50, and 26% average. The *n* was reduced due to the two weakest samples achieving below *P*_f_ = 0.03.

Differences in *n* result from higher gradients of low and high fiber or filler density areas [27], i.e., an increased anisotropy within a panel or between different panels. For the 0.44 mm data set, the two lowest *a*_uc_ samples probably had more lower fiber density or abrupt fiber density reduction sites than the other samples, which may be an explanation for the high variance. Shortening the glass fibers by extended mixing increased the *a*_uc_ and overcame this issue.

### 3.3. Optical Microscopy

**Figure 5:**
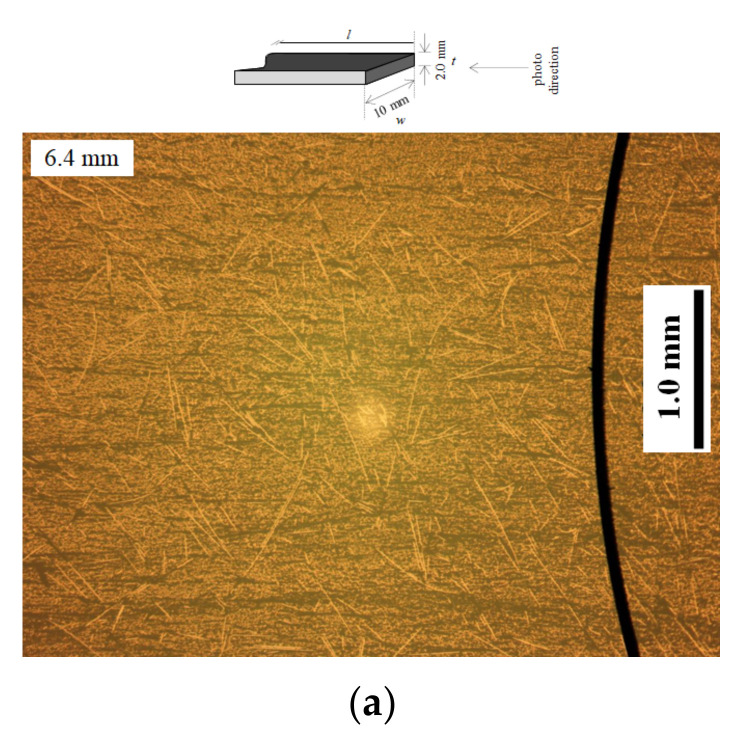

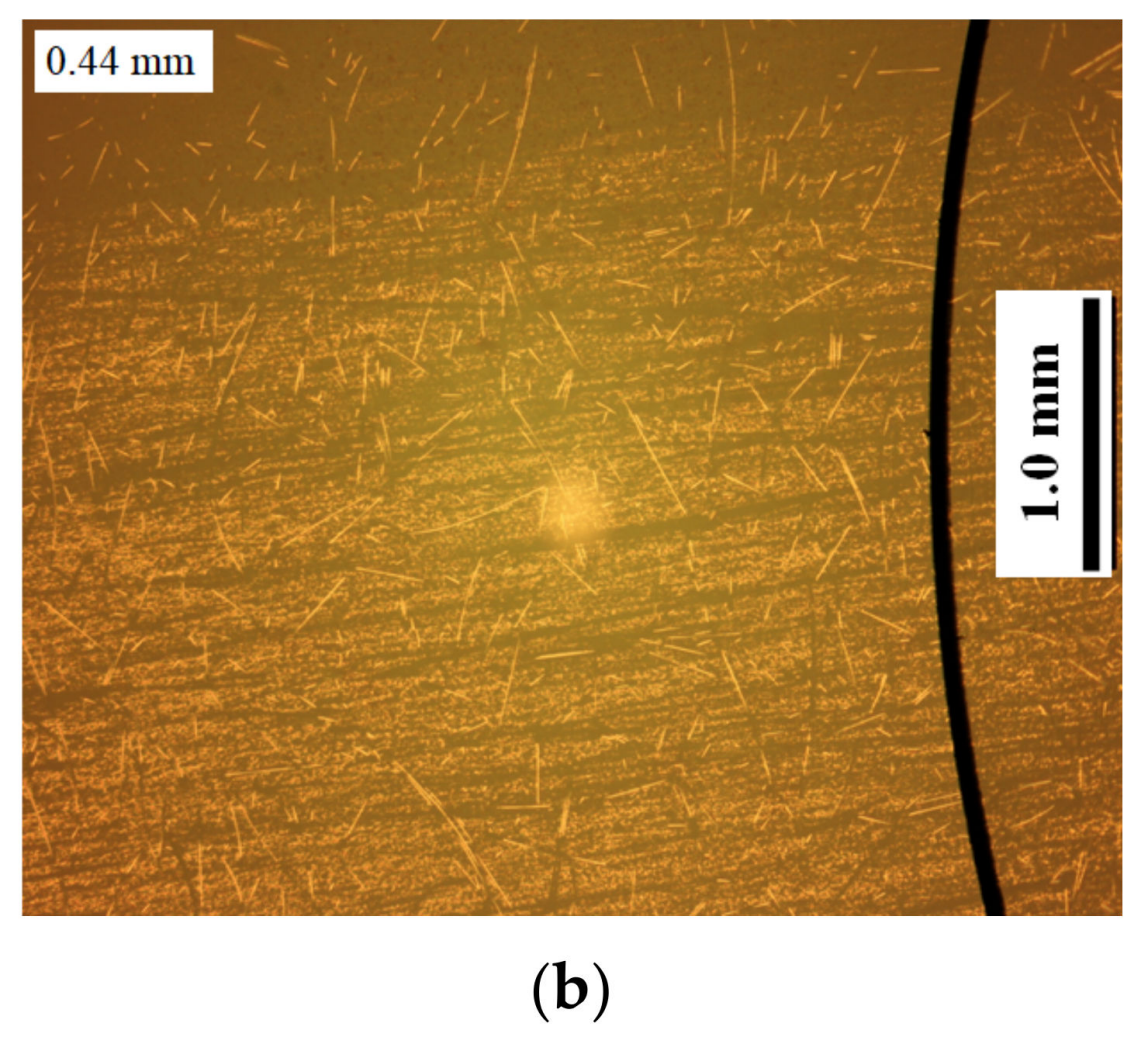
Optical microscope photos from center A-sections for: (**a**) nominal 6.4 mm, and (**b**) 0.44 mm BMC samples, respectively, and a diagram of optical microscopy direction.

Figure 5a,b shows photos by optical microscope of polished samples from center A-Sections for (a) nominal 6.4 mm, and (b) shortened fiber formulations, respectively. Photos were taken normal to sample cross-sections as illustrated. The extended mixing formulation appears to have a significantly higher proportion of shorter fiber cross-sections with higher, more homogeneous dispersion than the nominal 6.4 mm commercial fibers, allowing for an increased number of spaces between fibers, *S*_f_.

Note that the top photo of the nominal 6.4 mm specimen (Figure 5a) has curved long fibers probably configuring into the 2 mm panel thickness, which is mostly absent in the shorter fiber photo (Figure 5b).

### 3.4. Effect of Shortening Fibers on Impact Strength as a Function of Texture Angle

#### 3.4.1. [*θ*_t_ = Random (45) 0 to 90 deg] A-Section

**Figure 6:**
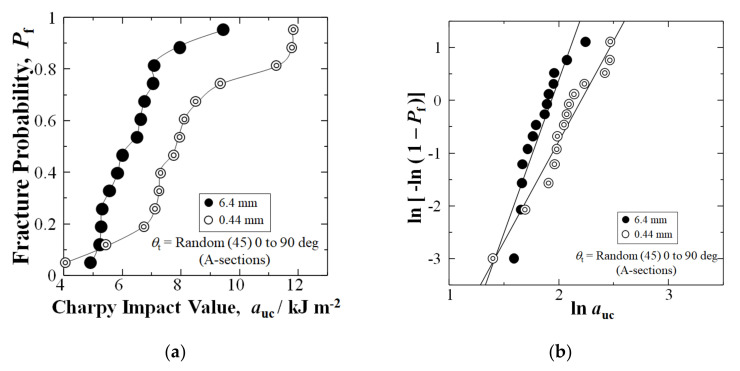
(**a**) Relationship between auc and Pf for nominal 6.4 mm and extended mixed 0.44 mm fiber samples, respectively, for A-Sections with θt of random (45) 0 to 90 deg between mold flow and longitudinal direction of testing sample; (**b**) Weibull 2-parameter plots of data in (**a**).

**Figure 7:**
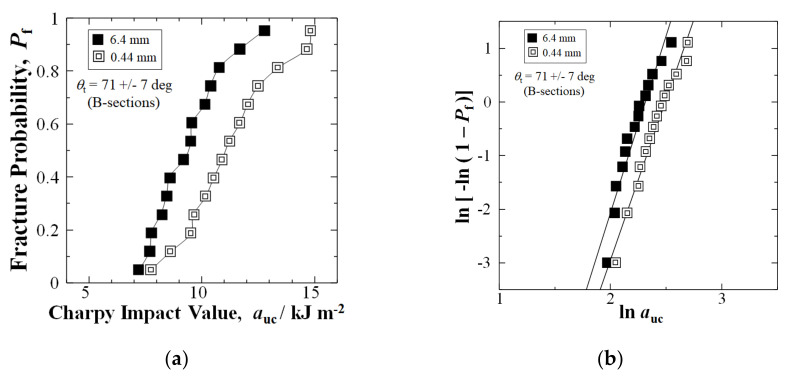
(**a**) Relationship between auc and Pf for nominal 6.4 mm and extended mixed 0.44 mm fiber samples, respectively, for B-Sections with θt of random (45) 0 to 90 deg between mold flow and longitudinal direction of testing sample; (**b**) Weibull 2-parameter plots of data in (**a**).

**Figure 8:**
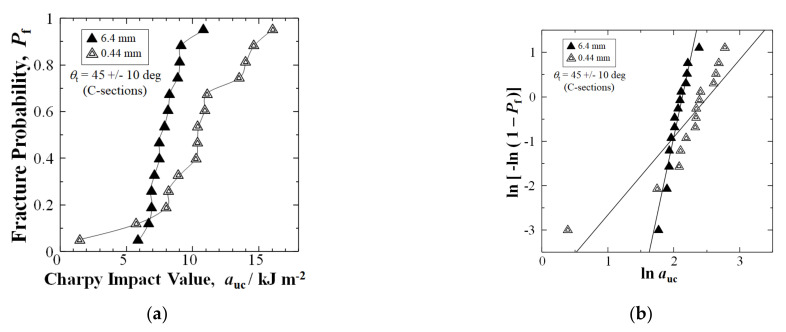
(**a**) Relationships between *a*_uc_ and *P*_f_ for nominal 6.4 mm and extended mixed 0.44 mm fiber samples, respectively, for C-Sections with *θ*_t_ of 45 +/− 10 deg between mold flow and longitudinal direction of testing sample; (**b**) Weibull 2-parameter plots of data in (**a**).

**Figure 9:**
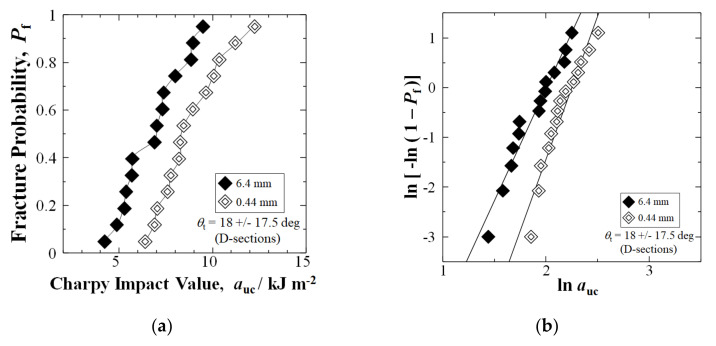
(**a**) Relationship between *a*_uc_ and *P*_f_ for nominal 6.4 mm and extended mixed 0.44 mm fiber sample, respectively, for D-Sections with *θ*_t_ of 18 +/− 17.5 deg between mold flow and longitudinal direction of testing sample; (**b**) Weibull 2-parameter plots of data in (**a**).

To show the effects of shortening fibers on the increase of impact strength according to the solidification texture angle, *θ*_t_, between mold flow and longitudinal direction of the testing sample, sections A, B, C, D that were depicted in the *P*_f_ vs. *a*_uc_ plot of Figure 2 are examined separately in Figure 6, Figure 7, Figure 8 and Figure 9, for 6.4 and 0.44 mm data sets, respectively.

Figure 6a shows that for the typically weakest center of the GFRP panel with a texture angle of random (45) 0 to 90 deg (A-sections) [27], shortening fibers by extended mixing improved the Charpy impact value of *a*_uc_ by 25%, from 6.26 to 7.86 kJm^−2^, at a median-fracture probability of *P*_f_ = 0.50. Moreover, at high *P*_f_ = 0.88, the *a*_uc_ improved remarkably by 48%, from 7.97 to 11.79 kJm^−2^. Figure 6a shows that the *a*_uc_ improved at all fracture probabilities, *P*_f_, above 0.05.

Furthermore, the extended mixing to 0.44 mm increased the average *a*_uc_ in A-Section by 28% over the 6.4 mm fiber length samples, from 6.41 (1.24) to 8.18 (2.27) kJm^−2^. Figure 6b shows that the Weibull modulus, *n* was reduced by extended mixing from 5.81 to 3.81, due to higher scatter in the 0.44 mm data set.

#### 3.4.2. [*θ*_t_ = 71 ± 7 deg] B-Section

Similar to the highest solidification texture angle, with a *θ*_t_ of 71 ± 7 deg (B-sections) in the GFRP panel, Figure 7a shows that shortening fibers to 0.44 mm by extended mixing improved the Charpy impact value *a*_uc_ by 18%, from 9.36 to 11.07 kJm^−2^, at a median-fracture probability of *P*_f_ = 0.50. Moreover, at high *P*_f_ = 0.88, the *a*_uc_ improved by 25%, from 11.70 to 14.67 kJm^−2^. Figure 7a shows that in B-Section the *a*_uc_ improved at all fracture probabilities, *P*_f_.

In addition, the average *a*_uc_ of 0.44 mm fiber length samples improved 19% above the 6.4 mm samples, from 9.44 (1.61) to 11.25 (2.11) kJm^−2^. Weibull modulus, *n*, slightly decreased for B-Section, from 6.61 to 5.96, as shown in Figure 7b.

#### 3.4.3. [*θ*_t_ = 45 ± 10 deg] C-Section

For the diagonal texture angle 45 ± 10 deg (C-sections), as shown in Figure 8a, shortening the fibers improved the *a*_uc_ by 35%, from 7.68 to 10.37 kJm^−2^ at a median-fracture probability of *P*_f_ = 0.50. Moreover, at high *P*_f_ = 0.88, *a*_uc_ improved remarkably by 60%, from 9.14 to 14.61 kJm^−2^.

Moreover, the average *a*_uc_ for C-Section improved by 30% over the 6.4 mm fiber length samples from 7.89 (1.28) to 10.25 (3.79) kJm^−2^.

The Weibull calculation in Figure 8b shows the *n* value reduced from 6.96 to 1.75, for the two weakest samples below *P*_f_ = 0.15. However, the *a*_uc_ increased at all *P*_f_ above 0.15.

#### 3.4.4. [*θ*_t_ = 18 ± 17.5 deg] D-Section

For the lowest solidification texture angle, namely, 18 ± 17.5 deg (D-sections), Figure 9a shows extended mixing improved the Charpy impact value *a*_uc_ by 20% over the 6.4 mm fiber length samples, from 6.96 to 8.36 kJm^−2^ at a median-*P*_f_ = 0.50; and at *P*_f_ = 0.88, the *a*_uc_ improved by 25%, from 8.95 to 11.21 kJm^−2^. In D-Section, the *a*_uc_ improved at all fracture probabilities, *P*_f_.

Moreover, the average *a*_uc_ of D-Section improved due to extended mixing, being 29% above the 6.4 mm fiber length samples, from 6.79 (1.65) to 8.79 (1.72) kJm^−2^.

Figure 9b shows that the Weibull modulus, *n*, improved slightly by extended mixing, from 4.50 to 5.76.

Interestingly, Figure 6, Figure 7, Figure 8 and Figure 9 show that the highest increase in *a*_uc_ was at a high-*P*_f_ of 0.88 for A-, B-, and C-Sections (48, 25, 60%), respectively, with D-Section also showing a significant increase of 25%. This could be explained by higher fiber density, *ρ*_f_., from the flows during compression and solidification.

Comparison of the four sections A, B, C, and D themselves is considered beyond the focus of this study, due to the large existing amount of data and explanation required.

In summary, shortening glass fibers in GFRP-BMC from 6.4 to 0.44 mm by 30 min of extended mixing increased the *a*_uc_ at all solidification texture angles of *θ*_t_: 0 to 90 (random), 71, 45 and 18 deg (Sections A, B, C, D); in addition to the Sections A, B, C and D cumulatively.

### 3.5. Mechanism of Strengthening by CTE Difference

**Figure 10:**
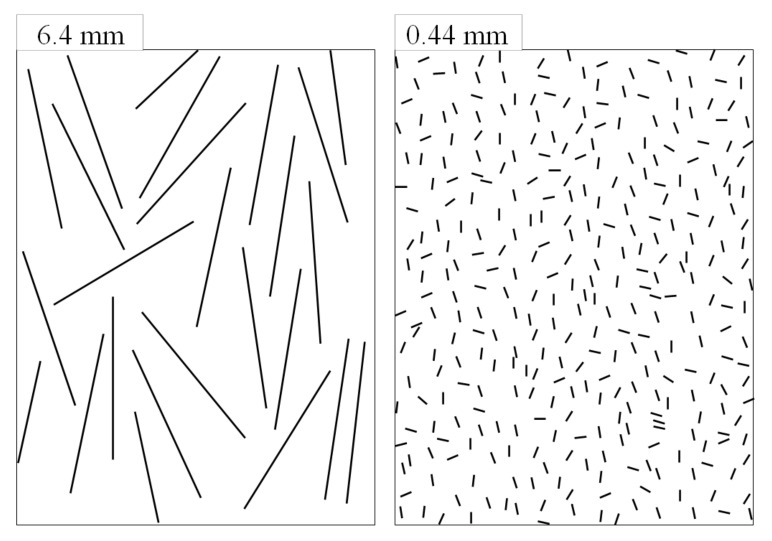
Schematic of increase in spaces between fibers, *S*_f_, showing closer fiber proximity. Total fiber length is illustrated as equal to represent a constant *V*_f_.

Figure 10 illustrates the action of the CTE difference between cured polyester resin matrix (55 to 100 × 10^−6^/K) [24] and E-glass fibers (5.4 × 10^−6^/K) [25] being the thermal residual stresses generated from the matrix to the fibers during cool down and shrinkage. As fiber length is shortened, the CTE difference acts in the increased number of spaces (*S*_f_) between fibers with closer proximity, collectively stiffening the composite and raising impact strength. To calculate the increase in *S*_f_ by shortening 6.4 mm fibers to 0.44 mm, fiber density, *ρ*_f_ (mm^−3^) is calculated by the following equation [8]:

*ρ*_f_ = *V*_f_/(π*r*^2^*l*_fiber_) (6)

where *r* is mean fiber radius taken to be 14 μm (0.014 mm), and *l*_fiber_ is mean fiber length (mm) [7]. This is assuming homogeneous fiber distribution and lengths. For 1 mm^3^ of composite, *ρ*_f_ designated |*ρ*_f_| will equal fiber number density, |*N*_f_| [8]:

*|ρ*_f_| = |*N*_f_| = |*S*_f_| (7)

which in turn is equal to number of spaces: the |*N*_f_| and |*S*_f_| are dimensionless quantities. This would hold true for fibers oriented parallel (0 deg) or any angle, *θ*, with respect to specimen length [8]. From this, |*S*_f_| increases in order of magnitude from 217 to 2950 mm^−3^, increasing the impact strength of the BMC.

### 3.6. All Texture Angles, θ_t_ Cumulative: Evaluation of Statistically Lowest Impact Value a_s_ at P_f_ = 0 Omitting 2 Lowest a_uc_ 0.44 mm Samples of P_f_ < 0.03

**Figure 11:**
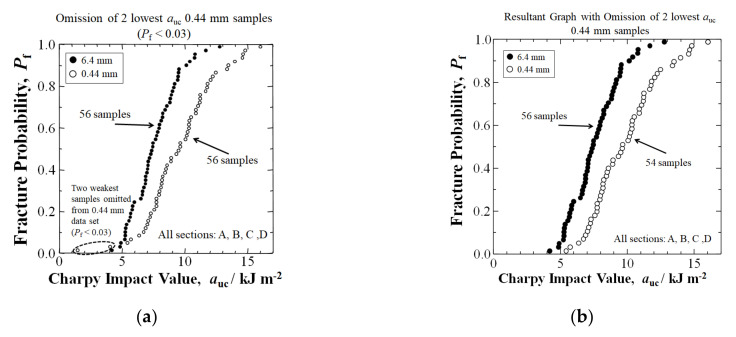
(**a**) Depiction of Figure 4a (all sections cumulative) indicating the 2 lowest *a*_uc_ samples (*P*_f_ < 0.03) to be omitted from the 0.44 mm data set for re-evaluation. (**b**) Resultant graph with omission of 2 lowest *a*_uc_ 0.44 mm samples, showing relationships between *a*_uc_ and *P*_f_.

In compression molding, complex flow patterns can result in the creation of high- and low- fiber density areas, the low fiber density area often lowering mechanical properties [27]. Figure 11a shows in the extended mixing 0.44 mm data set that there were two samples out of the total of 56 with a markedly lower *a*_uc_ (dotted oval) at the lowest *P*_f_ < 0.03, being evidence of significantly low fiber density areas. The two samples were omitted, thereby resulting in a 54 sample data set in Figure 11b. The number of nominal 6.4 mm fiber length samples remained unchanged at 56. The samples omitted from the 0.44 mm data set were C1′ and A2′ with an *a*_uc_ of 1.48 kJm^−2^ and 4.06 kJm^−2^, respectively. However, the adjoining specimens C2′ and A1′ had much higher *a*_uc_ at 8.19 and 9.35 kJm^−2^, respectively, being evidence of high density gradients created during the flow and solidification of the GFRP-BMC paste.

To assess safety, the statistically lowest *a*_s_ at *P*_f_ = 0 was calculated using 3-dimensional Weibull analysis. The *a*_s_ at *P*_f_ = 0 is useful for quality control (QC) of mass-produced parts. If the statistical equation is assumed to be applicable to the measured *a*_uc_ value, the *P*_f_ depends on the risk of rupture [35,37]. In predicting the required value for a new structural material, the *a*_s_, the coefficient, *m*, and the constant, *a*_III_, are key parameters. The equation is:

*P*_f_ = 1 − exp[−([*a*_uc_ − as]/*a*_III_)*^m^*] (8)

Rearranging in linear form yields:

ln(−ln(1 − *P*_f_)) = *m*ln(*a*_uc_ − *a*_s_) − *m*ln*a*_III_ (9)

**Figure 12:**
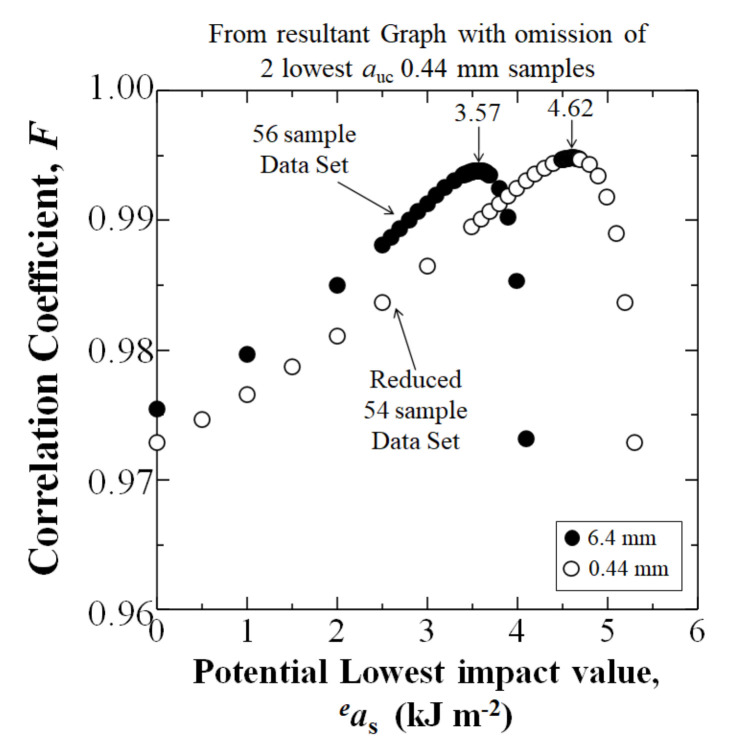
Iteration of potential impact value (*^e^a_s_*) to obtain the statistically lowest impact value *a_s_* at *P*_f_ = 0 by 3-parameter Weibull analysis with the omission of the 2 lowest *a*_uc_ 0.44 mm samples for the data in Figure 11b.

As shown in Figure 12, when the linear form in Equation (9) is iterated for the highest correlation coefficient, *F*, the *a*_s_ is obtained. When the two lowest *a*_uc_ samples were omitted from the 0.44 mm data set, the *a*_s_ at *P*_f_ = 0 for the adjusted 0.44 mm sample data set (4.62 kJm^−2^) was higher than the *a*_s_ of the commercial length of 6.4 mm (3.57 kJm^−2^), showing that an increased level of safety is possible.

## 4. Conclusions

This study demonstrates that in a highly CaCO_3_ filled 3-phase filler, fiber and polymer GFRP-BMC, impact strength can be increased by shortening glass fibers from the commercial fiber length of 6.4 mm (1/4 in) to 0.44 mm, by 30 min of extended mixture of the paste prior to compression molding, without any change in compression molding equipment. This has not been previously reported in the literature, and is opposite to 2-phase fiber and polymer systems where increasing the fiber length increases the mechanical properties. The strengthening can be explained by the homogeneous distribution of increased thermal compressive stress sites induced by the increased fiber number density generated by a difference in coefficient of thermal expansion (CTE) by the matrix on the glass fibers during cooling down and shrinkage. The higher number of spaces between fibers per mm^3^, |*S*_f_|, generated, is increased by an order of magnitude enhancing the impact energy. These results can be applied to maximize the safety of BMC materials through the prevention of impact damage caused by bird strike, volcanic rock, hailstones, or in space, by micrometeoroids and debris.

## Figures and Tables

**Table 1:** GFRP-BMC components.

Component	Mass %
propylene glycol maleate polyester	13.75
styrene butadiene copolymer	12.75
commercial E-glass fibers	11
CaCO_3_ filler	55
aluminum silicate filler	3
magnesium hydroxide	0.5
proprietary initiators and inhibitors	4

**Table 2:** Compression molding parameters.

Parameter	Condition
Mold Pressure	5.5–6.9 MPa (800–1000 psi)
Temperature	422 K (149 °C)
Cure Time	2 min
Mold Type	matched metal die compression mold
Panel Size	304.8 × 304.8 × 2 mm
*V*_f_ (E-glass fibers)	0.080
*V*_f_ (CaCO_3_ filler)	0.377
*V*_f_ (remaining polymer mixture)	0.543

**Table 3:** Fiber lengths.

Fiber Lengths	Mixing
6.4 mm (1/8 in)	20 min
0.44 mm [7,8]	20 min +additional 30 min

**Table 4:** Number of GFRP-BMC samples tested.

Fiber Length (mm)	Total A + B + C + D	A	B	C	D
6.4	56	14	14	14	14
0.44	56	14	14	14	14

**Table 5:** Average Charpy impact values, *a*_uc_, and standard deviations for all texture angles, *θ*_t_ cumulative (56 sample data set), and for each solidification texture angle sub-quadrant, A, B, C, and D. Standard deviations are in brackets.

Charpy Impact Values (kJm^−2^)					
Fiber Length(mm)	Total A + B + C + D	A	B	C	D
6.4	7.63 (1.85)	6.41 (1.24)	9.44 (1.61)	7.89 (1.28)	6.79 (1.64)
0.44	9.62 (2.80)	8.18 (2.27)	11.25 (2.11)	10.25 (3.79)	8.79 (1.72)
% imp	26.1	27.6	19.1	29.9	29.5

## Data Availability

The data presented in this study are available on request from the corresponding author. At the time the project was carried out, there was no obligation to make the data publicly available.
